# Cinnarizine versus Topiramate in Prophylaxis of Migraines among Children and Adolescents: A Randomized, Double-Blind Clinical Trial

**Published:** 2014

**Authors:** Mahmoud Reza ASHRAFI, Zeinab NAJAFI, Masih SHAFIEI, Kazem HEIDARI, Mansoureh TOGHA

**Affiliations:** 1 Pediatrics Centre of Excellence, Department of Pediatric Neurology, Children’s Medical Centre, Tehran University of Medical Sciences, Tehran, Iran; 2 Growth and Development Research Center, Tehran University of Medical Sciences, Tehran, Ira; 3 Sports Medicine Research Center, Neuroscience Institute, Tehran University of Medical Sciences, Tehran, Iran; 4 Department of epidemiology and biostatistics, School of Public Health, Tehran University of medical sciences, Tehran, Iran; 5 Iranian Centre of Neurological Research, Neuroscience Institute, Tehran University of Medical Sciences, Tehran, Iran; 6 Department of Neurology, Sina Hospital, Tehran University of Medical Sciences, Tehran, Iran

**Keywords:** Cinnarizine, Migraine, Pediatrics, Topiramate

## Abstract

**Objective:**

Migraines, a common health problem in children and adolescents, still do not have an FDA approved preventive treatment for patients under the age of 18 years. This study compares and contrasts the efficacy and safety of cinnarizine and topiramate in preventing pediatric migraines.

**Materials & Methods:**

In this randomized, double-blind clinical trial 44 migrainous (from 4–15 years of age) were equally allocated to receive cinnarizine or topiramate. The primary efficacy measure was monthly migraine frequency. Secondary efficacy measures were monthly migraine intensity and ≥ 50% responder rate. Efficacy measures were recorded at the baseline and at 4, 8, and 12 weeks of treatment.

**Results:**

During the double-blind phase of the study, monthly migraine frequency and intensity were significantly decreased in both the cinnarizine and topiramate groups when compared to the baseline. However, at the end of the study, the cinnarizine group exhibits a significant decrease from the baseline in the mean monthly migraine intensity when compared to the topiramate group (4.7 vs. 3, respectively; 95% CI = -0.8 to -3.2).

**Conclusion:**

No significant difference between cinnarizine and topiramate was found for the prevention of pediatric migraines. Both treatments were well tolerated.

## Introduction

Migraine is a common health problem in children and adolescents (1-3). It can negatively affect children and adolescents in their daily activities and school performances as well as causes school absenteeism (3,4). The mean age for the onset of migraines varies in children with gender, and is reported to be 7.2 years in boys and 10.9 years in girls 5, 6. The prevalence of migraine headaches among children and adolescents aged between 5 to 15 years ranged from 2.7–10.6% 3. The prevalence increases with age and is reported to be up to 28% in adolescents, aged 15 to 19-years 3, 7. Migraine headaches show a male predominance in children, which shifts to a slight female predominance in adolescence that continues into adulthood (3, 5).

Management of migraine headaches in children and adolescents consists of bio-behavioral treatments (including life style changes, stress management, and bio-feedback strategies), acute treatments, and preventive treatments 8). Preventive management is recommended when the frequency of migraine attacks is three or more per month or the migraine attacks are significantly disabling (as assessed by a scoring system such as the Pediatric Migraine Disability Assessment Scale) (9, 10). No medications are currently approved by the Food and Drug Administration (FDA) for the preventive treatment of migraine headaches in patients under the age of 18 years (8). The preventive treatment of migraines in children and adolescents is based on information extracted from adult trials on migraines.

Topiramate has been approved for use of migraine prevention in adults in Europe and by the FDA (11). Two randomized, double-blind, placebo-controlled studies show the effectiveness of topiramate in significant reduction of monthly migraine frequency in children and adolescents (12,13). In another randomized, double-blind, placebo-controlled trial, topiramate effectively reduced the mean of monthly migraine frequency in children and adolescents; however, it didn’t reach significance and the results trend was towards significance (14). According a pooled analysis of three pivotal trials, topiramate might reduce migraine frequency in adolescents (15). Some other uncontrolled studies also showed the effectiveness of topiramate in reducing monthly migraine frequency in children (16-19). The frequency of side effects varied considerably among previous studies with the most frequent side effects reported as weight loss, anorexia, abdominal pain, sedation, paresthesia, and difficulties in concentration (12-19).

The limited effectiveness of cinnarizine as a preventive treatment of migraine headaches in adults is known. Two open-label trials and a randomized, double-blind clinical trial has shown the effectiveness of cinnarizine treatment in reducing the monthly migraine frequency in adults (20-22). These studies also reported no serious adverse effects (20-22). To the best of our knowledge, no study has yet investigated the effectiveness of cinnarizine treatment as a prophylaxis for migraine headaches among children and adolescents.

To the best our knowledge, no study has compared the efficacy and safety of cinnarizine with that of topiramate for migraine prevention among children and adolescents. In this regard, we conducted a randomized, doubleblind clinical trial to evaluate the efficacy and safety of cinnarizine in comparison to topiramate as preventive treatments for migraine headaches among children and adolescents.

## Materials & Methods

We conducted a randomized, double-blind comparative trial composed of a prospective baseline lasting for 4 weeks followed by a double-blind phase lasting for 12 weeks. 

Patients from 4–17 years of age who were admitted to the pediatric neurology clinic of Children’s Medical Center Hospital affiliated with Tehran University of Medical Sciences with the complaint of headache or diagnoses for migraines were evaluated. A complete history of the patients’ migraine characteristics as well as a complete general medical history was recorded along with a general and neurological physical examinations were performed. The information collected from recording the medical history of patients and their physical examinations, patients who met the eligibility criteria (including inclusion and exclusion criteria) entered the prospective baseline phase of the study. 

The inclusion criteria were as follows: 

1. Children and adolescents, Aged 4–17 years, diagnosed with migraines (with or without aura) according to the International Headache society criteria (23);

2. Having experienced one or more migraine attacks per month or severe dysfunction in daily and school activities; and/ or,

3. Children and adolescents without any known structural brain lesions or other systemic conditions causing the headaches.

The exclusion criteria were as follows: 

1. Diagnosis of chronic headache, complications of migraine or migraine variant;

2. Focal neurologic deficit;

3. Severe adverse effects related to the study treatment drugs that are listed in the contraindications at the beginning or during the double-blind phase of the study;

4. Known concomitant serious disease (hepatic, renal, cardiovascular, or thyroid disease); and/ or,

5. Use of prophylactic migraine therapy in at least one preceding month.

In the 4 weeks prospective baseline, the previous medications of patients for migraines, either for preventive or acute treatment, were halted; and the frequency and intensity of the migraines were recorded. Each patient was given a diary to record the frequency and intensity of each migraine. Patients had to fill out the diary every day, whether they experienced migraine or not. If they had migraine, they also filled out a checklist of headache characteristics attached to the diaries. 

Patients who completed the prospective baseline phase of the study entered the 12 weeks double-blind phase of the study. At the start of the double-blind phase, patients were randomized into two treatment groups. One group of participants received cinnarizine as the preventive treatment for migraines (the cinnarizine group); and the other group of participants received topiramate as the preventive treatment for migraines (the topiramate group). The study had no placebo group. Since the intractable nature of the migraine headaches of patients, it was immoral to have a group of participants receiving no preventive treatment. In order to maintain double blinding, each patient was given an ID code and the drugs were given to the patients using their ID codes. Cinnarizine and topiramate tablet characteristics including the shape, color, and drug packages were similar to each other but not the same). 

At the start of double-blind phase of the study, a blood count test and a liver enzyme test (SGOT) were performed. The tests were re-performed at the end of 12 weeks of the double-blind phase. 

In the double-blind phase of the study, there were two groups of patients: 1. the cinnarizine group; and 2. The topiramate group. The cinnarizine group was administrated with a dose of 37.5 mg every day for patients aged 4–11 years; and 50 mg every day for patients aged 12–17 years, from the beginning of the double blind phase to the end of the 12 weeks. The topiramate group was administrated with a dose of 50 mg every day from the beginning of the double blind phase to the end of the 12 weeks. Adjustment of the dose of cinnarizine and topiramate in presence of intolerability or occurrence of serious side effects related to the treatment drugs could be considered due to the neurologist of this study’s permission. In addition, patients were permitted to take analgesics for abortive treatment of acute migraine attacks throughout the study.

Patient information about the characteristics of migraine attacks (including the frequency and intensity of attacks) during the double-blind phase was recorded using diaries. Each patient was provided with a diary for 90 days, in which all migraine attack characteristics consist of the duration in hours and the intensity of attacks were recorded. Parents were also involved by advising them to assist their children to correctly fill out the diaries. 

Follow up visits were scheduled at 4, 8, and 12 weeks during the double-blind phase of the study. At each visit, the diaries were checked and collected. Patients were evaluated using detailed questionnaires to investigate the occurrence of the side effects during the last 4 weeks (the gaps between visits) and the relation of the side effects to the treatment drugs. In this regard, the frequency and the occurrence of treatment related side effects were assessed.

**Efficacy measures**

To measure the efficacy of cinnarizine and topiramate treatments, the frequency and intensity of migraine attacks and 50% responder rate to the treatments were evaluated. All required data for calculating the intended measures were based on information obtained from the diaries.

Frequency of migraine attacks was defined as the mean number of migraine attacks that fulfilled the IHS criteria for migraine with or without aura 23 per each 4 week period.

The intensity of attacks was measured using the Visual Analogue Scale (VAS). This scale consists of a 10 cm line that is divided into 10 parts, which are numbered 0–10. Zero indicates no pain and 10 indicates the worst pain imaginable. Migraine intensity was defined as the mean intensity of migraine attacks per each 4 week period.

A 50% responder rate was defined as the percentage of patients who had a migraine frequency that was reduced greater or equal to 50%.

**Safety measures**

At each visit of the double-blind phase, safety of the treatment drugs was assessed by asking the patients’ history of side effects occurrence during the last 4 weeks with a detailed questionnaire. The relation of the side effects to the treatment drugs was also assessed at each visit by interviewing the patients. Special attention was paid to the occurrence of sleepiness, decreased appetite, and weight loss.

**Statistical analysis**

Evaluating the efficacy and safety of the treatments was based on information obtained from the diaries, patient history (which was recorded at start of the double-blind phase and the visits during the double-blind phase), general and neurological examinations, and laboratory tests (including, blood count and liver function tests). Average descriptive statistics and standard deviations were provided for the treatment groups separately and for the total population. The differences between the treatment groups’ baseline characteristics were assessed by using two sample (unpaired) t test. The comparison between the baseline phase values and 4, 8, and 12 weeks of treatment during the double-blind phase values was performed using sample (paired) t test. In order to analyze the treatment comparability, a student’s t test for independent samples and analysis of variance with repeated measures over time was used. Results are expressed as a mean and p<0.05 was considered statistically signiﬁcant. Data were analyzed using SPSS software (ver. 18) and conﬁdence interval analysis software.

This trial was approved by the ethics committee of Tehran University of Medical Sciences. All patients were given informed consent about the study prior to entering the study.

## Results

A total of 40 participants (23 male and 17 female) enrolled in the study, with a mean age of 9.0 (range, 4–15) years. At the baseline, the mean (SD) of monthly migraine frequency was 7.7±7.2 and the mean (SD) of monthly migraine intensity was 6.9±2.3 (due to VAS scaling). Participants were randomly allocated into two treatment groups (cinnarizine n=20; topiramate n=20). There were no statistically significant differences between the treatment groups regarding the participant age, the baseline mean of monthly migraine frequency, and the baseline mean of monthly migraine intensity (p= 0.46, p = 0.81, and p = 0.30, respectively) (Table 1) represents demographic data and baseline characteristics of the study participants.

**Table1:** Demographic Data and Baseline Characteristics of Participants

	**Cinnarizine**	**Topiramate**
Age,		
mean ±SD, year range, year	9.3 ±2.43 5-13	8.7±3.03 4-15
Gender, n (%)		
Male Female	12 (60) 8 (40)	11 (55) 9 (45)
Mean migraine frequency (per month) ±SD	8.0 ±7.98	7.5 ±6.43
Mean migraine intensity (per month) ±SD	7.3±2.12	6.5 ±2.42

**Efficacy measures**

After 4 weeks of treatment, 35% of the cinnarizine group participants and 30% of the topiramate group participants showed 50% responder rate; which was statistically significant for both groups (p = 0.007, 95% CI 0.70-3.90; p = 0.008, 95% CI 0.57-3.33; respectively). A significant reduction in the mean of monthly migraine intensity for cinnarizine (p < 0.001, 95% CI 1.15-2.25) and topiramate (p = 0.002, 95% CI 0.50-1.90) groups was also found as opposed to the baseline values. 

At the second visit during the double-blind phase (week 8), statistically significant changes were also observed. Both treatment groups showed a statistically significant 50% responder rate (cinnarizine: 55%, p = 0.004, 95% CI 1.26-5.74; topiramate: 50%, p = 0.001, 95% CI 1.70- 5.40). The same was found for the mean of monthly migraine intensity (cinnarizine: p < 0.001, 95% CI 2.10- 3.90; topiramate: p < 0.001, 95% CI 1.24-3.16). After 8 weeks of treatment compared with the baseline values, reduction of monthly migraine frequency, and intensity demonstrated no statistically significant differences between the cinnarizine and topiramate groups (p> 0.05). 

The mean of monthly migraine frequency and intensity at the end of the 8th week of treatment in comparison to the end of the 4th week significantly lowered within treatment groups (p <0.05), but no significant differences were found for the cinnarizine versus the topiramate group(s) (p> 0.05). At the last visit during the double-blind phase (week 12), 85% of the cinnarizine group and 65% of the topiramate group were associated with significant 50% responder rate (p= 0.001, 95% CI 2.88-9.12; p= 0.001, 95% CI 2.18-7.32; respectively). The reduction in the mean of monthly migraine intensity was also significant for both groups (cinnarizine: p < 0.001, 95% CI 3.67-5.73; topiramate: p <0.001, 95% CI 1.80-4.20) compared with the baseline values. The reduction of monthly migraine frequency showed no significant differences for the cinnarizine group versus the topiramate group (p> 0.05), whereas monthly migraine intensity in the cinnarizine group reached a significant reduction in comparison to the topiramate group (p < 0.05). During the last 4 weeks of treatment, the reduction in monthly migraine frequency and intensity was significant for both treatment groups (p<0.05); however, these values demonstrated no differences between the cinnarizine and the topiramate group (p> 0.05). 

Table 2 shows the mean of monthly migraine frequency and intensity in the baseline phase and during the doubleblind phase (after 4, 8, and 12 weeks of treatment) regarding the treatment groups.

Figure(s) 1 and 2 show the reduction in the mean of monthly migraine frequency and intensity from the baseline through the double-blind phase compared to the responses to the cinnarizine and the topiramate treatments.

**Fig 1:**
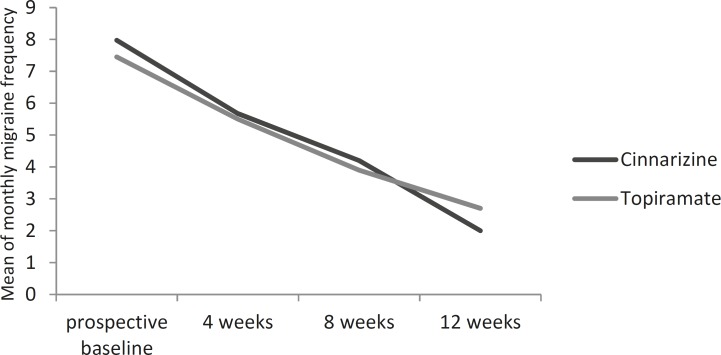
Mean of monthly migraine frequency over time cinnarizine versus topiramate

**Fig 2:**
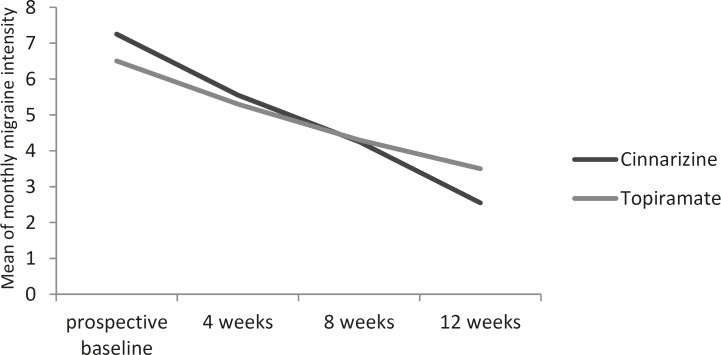
Mean of monthly migraine intensity over time cinnarizine versus topiramate

**Table2:** Reduction in Monthly Migraine Frequency and Intensity Over Time that Compares the Treatment Groups

	**Cinnarizine** **(N = 20)**	**Topiramate** **(N = 20)**
Prospective baseline period Migraine frequency, (per month) Mean±SD Migraine intensity, (per month) Mean ±SD	8.0 ± 7.98 7.3 ± 2.12	7.5 ± 6.43 6.5 ± 2.42
After 4 weeks of double-blind phase Migraine frequency, (per month) Mean±SD P (vs. baseline) Migraine intensity, (per month) Mean ±SD P (vs. baseline)	5.7 ± 5.70 0.007 5.6 ± 2.01 < 0.001	5.5 ± 6.02 0.008 5.3 ± 2.77 0.002
After 8 weeks of double-blind phase Migraine frequency, (per month) Mean±SD P (vs. baseline) Migraine intensity, (per month) Mean ±SD P (vs. baseline)	4.5 ± 4.57 0.004 4.3 ± 2.15 < 0.001	3.9 ± 3.91 0.001 4.3 ± 2.62 < 0.001
After 12 weeks of double-blind phase Migraine frequency, (per month) Mean±SD P (vs. baseline) Migraine intensity, (per month) Mean ±SD P (vs. baseline)	2.0 ± 2.47 0.001 2.6 ± 2.37 < 0.001	2.7 ± 3.26 0.001 3.5 ± 2.74 < 0.001

P values are measured using sample (paired) *t* test, from the comparison between baseline phase values and 4, 8, and 12 weeks of treatment during the double-blind phase values.

**Safety measures**

Mild treatment related adverse effects were reported in 4 subjects of the cinnarizine group, which was mild sleepiness. In the topiramate group, 6 subjects experienced mild treatment related adverse effects that included mild sedation (71.4%) and mild appetite decrease (14.3%). One subject in the topiramate group had serious adverse effects, which were severe decrease in appetite and weight loss (2 Kg, after 1-month treatment). The serious adverse effects did not result in withdrawal, but adjustment of the dose of topiramate. In total 27.5% of subjects (11/40) experienced mild treatment related adverse effects of which the most common was sedation (22.5%) (Table 3). No life-threatening side effects were reported during the double-blind phase in both treatment groups.

The analysis revealed no statistically significant differences between the cinnarizine group and the topiramate group for treatment related adverse effects (p= 0.29).

**Table 3:** Incidence of Adverse Effects During the Ddouble-blind Phase for the Treatment Groups

**Adverse effect**	**Cinnarizine** **(N = 20)** **n(%)**	**Topiramate** **(N = 20)** **n(%)**
Mild sedation	4 (20)	5 (25)
Sever sedation	0 (0)	0 (0)
Mild appetite decrease	0 (0)	1 (5)
Sever appetite decrease	0 (0)	1 (5)
Weight loss	0 (0)	1 (5)

## Discussion

In the current study, we evaluated the frequency and intensity of monthly migraine attacks in children and adolescents aged 4–17 years before and after (during a 12 weeks treatment period) receiving the cinnarizine or the topiramate treatment; and compared the results of the treatment groups with each other. We also assessed the responder rate to cinnarizine and topiramate treatment by comparing with the baseline and every 4 weeks of treatment (up to 12 weeks). In addition, frequency of probable adverse effects related to the cinnarizine and the topiramate treatment and the safety of the treatments were evaluated.

We demonstrated that the cinnarizine and topiramate treatments resulted in significant reductions in the frequency and intensity of monthly migraine attacks from the baseline through 12 weeks of treatment. The reduction in the mean of monthly migraine frequency and intensity showed no significant differences for cinnarizine versus topiramate treatments from the baseline through 12 weeks of treatment with exception that cinnarizine was significantly more effective than topiramate in the reduction of monthly migraine intensity for the last 4 weeks of double-blind phase compared with the baseline. The responder rate (50%) was also significant for both treatment groups from the baseline through 12 weeks of treatment. With regard to the safety measures, both treatments were well tolerated and no life-threatening side effects were reported. 

Two previous controlled trials and a pooled analysis of three pivotal trials have been reported in this study and the significant effect of topiramate to reduce monthly migraine frequency (12, 13, 15). Another controlled trial has reported that the topiramate effect in the reduction of monthly migraine frequency was not significant; however, the trend of the results was toward significance 14. A number of uncontrolled trials also supported the idea of the effectiveness of topiramate in the reduction of monthly migraine frequency (16-19).

A reported 50% responder rate to the topiramate treatment varies in different studies, from 43.1–95.2% (12-14,17). There are some studies in Iran that show the efficacy and safety of topiramate for the prophylaxis of childhood migraines (24, 25). In the current study, 50% responder rate was reported to be 30%, 50%, and 65% for the duration of treatment with topiramate (4, 8, and 12 weeks, respectively). This variety probably derives from the differences in administrated topiramate doses, duration of treatment with topiramate, and the baseline frequency of patient migraine headaches (13, 14, 26). 

The effectiveness of topiramate on the reduction of monthly migraine intensity has been reported by different studies (12,16-18). Two studies measured the migraine intensity by using Pediatric Migraine disability assessment score (PedMIDAS) (12,18), which is incomparable to our study intensity values, because our measurement for migraine intensity used VAS. Hershey et al used VAS scaling for the assessment of migraine intensity and reported significant reductions in migraine intensity 17 with the same found in our study. 

The frequency of side effects related to topiramate administration varied considerably among studies from 14–81% (measured as the percentage of participants that experienced at least one adverse effect during the treatment period of the study) (12, 13, 16-18). The most frequent reported side effects were weight loss, anorexia, abdominal pain, sedation, paresthesia, and difficulties in concentration (12-19). Withdrawal from the study caused by adverse effects was low (13, 14). We reported that the frequency of the side effects was 30% and the most frequent side effects were sedation and appetite decrease among the participants of the topiramate group. 

No dropouts occurred in our study due to adverse effects. The variety in the frequency of adverse effects among different studies may be due to the administrated dose of topiramate. In total, topiramate was well tolerated by patients in previous studies (13-18) as in this study. 

Although, further controlled studies should be carried out to assess the frequency of side effects of topiramate as preventive treatment of migraine headaches with regard to the different daily doses of topiramate.

To the best of our knowledge, the current study is the first study that investigates the effectiveness of cinnarizine treatment as a prophylaxis of migraine headaches among children and adolescents. In this regard, the results of our study on the effectiveness and safety of cinnarizine as a preventive treatment of migraine headaches among children and adolescents are incomparable with the limited data of previous studies on the effectiveness of cinnarizine in migraine prevention among adults (20-22).

A limitation of our study was that it had no placebo group. Since our probable participants who met the primary criteria to enter the baseline period of the study had migraine characteristics that make it impossible to manage migraines with using no preventive medications.

It was immoral to randomize them into an additional group in which the participants would receive a placebo. 

In this regard, further studies evaluating the effectiveness of cinnarizine and/or topiramate on migraine prevention in comparison to a control group (which is receiving a placebo) should be conducted. 

There is a lack of evidence in the literature about the effectiveness and safety of cinnarizine treatment either in children and adolescents or in adults. In addition, there is no approved medication yet available for migraine prevention in children and adolescents. Further open-label and controlled trials should be conducted on investigating the effectiveness and safety of cinnarizine treatment in migraine prevention with special regard to children and adolescents.

**In conclusion, **both cinnarizine and topiramate treatments demonstrated efficacy in the prevention of migraine headaches in children and adolescents. Cinnarizine can be considered as an effective and novel preventive treatment for pediatric migraines. Overall, both treatments were well-tolerated and safe.
